# Rectal-cancer radiotherapy damages the perineal muscle floor

**DOI:** 10.1093/gastro/goaa037

**Published:** 2020-06-26

**Authors:** Luca Reggiani Bonetti, Antonio Manenti, Graziana Gallo, Federica Domati

**Affiliations:** g1 Department of Pathology, Polyclinic Hospital, University of Modena, Modena, Italy; g2 Department of Surgery, Polyclinic Hospital, University of Modena, Modena, Italy

The interesting contribution of Qin *et al*. [1] about radiation injury on the rectum deserves to be integrated with observations regarding the equivalent damage to the perineal striated muscles. In approaching this topic, not yet extensively studied, we were interested at first in delineating its basic pathology by histological methods, as already proposed [2, 3]. For this, we selected from our anatomopathological archive 20 surgical specimens of abdominoperineal resections. In the first group, we enclosed 10 surgical specimens of lower rectal cancers operated on 5–6 weeks after a neoadjuvant radiochemotherapy, consisting of 50 Gy, fractioned in 5 weeks, and associated with a FOLFOX pharmacological treatment. In the second group, we included 5 cases of the same pathology, at a lower stage, and submitted for surgery 10 days after a ‘short-term’ neoadjuvant radiotherapy of 25 Gy in 5 fractions over 1 week. A third group encompassed 5 surgical specimens of abdominoperineal resection, performed for recurrent anal cancer 1 year after the same radiation treatment of 50 Gy as applied in the first group. From our study, we excluded cases with neoplastic infiltration of the perineal muscles, previous perineal surgery, radiation or trauma, systemic diseases such as diabetes, vasculitis, connective-tissue or musculoskeletal disorders, manifest atherosclerosis, morbid obesity, and poor nutrition. Clearly, the second and third groups were implemented for dose- and time-related controls, in comparison to the first group. The perineal striated muscles found in the specimens were extensively studied with serial histological sections of 4 mm and different staining techniques (Haematoxylin-Eosin, Trichrome Masson, and Mallory Azan); immunohistochemical analyses were performed with anti-Sarcomeric Actin antibodies (clone alpha-Sr-1, 1:20 dilution).

The histological results of the first group, which we considered ‘short-term’, demonstrated in about 30% of all the myocytes nuclear pycnosis and karyorhexis, in the cytoplasm disappearance of the characteristic striated bands and a decreased actin affinity to actin, until a homogeneous and amorphous feature. Similarly, the extracellular matrix was extensively disrupted in both its cellular and fibrillary components. A similar degenerative process involved a large number of arterioles and metarterioles, where the nuclei of the endothelial cells became prominent, the internal elastic lamella was disrupted, and the smooth muscle cells of their medial layer were replaced by collagen fibers and activated fibroblasts. Their increased permeability caused erythrocyte diapedesis and interstitial edema, entailing dilatation of the lymphatic capillaries (Figure 1A–C). In the second group, corresponding to cases of ‘short-term’ radiotherapy, no important histological lesions were found. In contrast, in the third group, corresponding to ‘long-term’ observations and referring to cases submitted for surgery 1 year after a radiotherapy treatment of 50 Gy, we observed a diffuse interstitial fibrosis, consisting of a dense connective tissue that wrapped the irradiated muscles and penetrated inside the perimysium, so altering the layout of the myofibers, appearing no longer assembled in distinct fascicles. The corresponding myocells acquired a spindle-like shape. The peripheral nervous fibers too were wrapped in the same fibrotic connective tissue; the lymphatic channels persisted as dilated (Figure 1D–F). A sharp band-like demarcation with the muscles outside the radiation field was evident in the specimens of the first and third groups.

**Figure 1.**
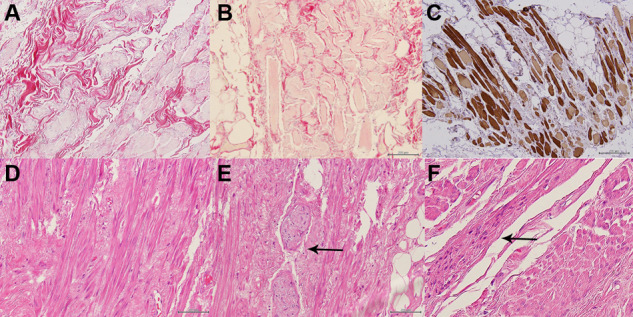
Histology panels of radiation injury (20×). At ‘short-term’ (Trichrome Masson): diffuse interstitial edema, myocell cytoplasms pale-stained (A) and (B) and a decreased affinity of the striated bands with actin (C). At ‘long-term’ (Haematoxylin-Eosin): new spindle-shaped cells (D), with evident interstitial fibrosis wrapping also the peripheral nerves (E, arrow) and a cluster of activated myoblasts (F, arrow).

As first interpretation, we remarked the evident connections between the detected lesions and the radiation dose or the time elapsed before surgery. In particular, in the ‘short-term’ results, we considered the muscle pathology as correlated with a process of hyaline degeneration, progressively leading to a cellular coagulative necrosis and electively deteriorating the more complex proteins, the nuclear DNA, and the cytoplasmic filaments of actin and myosin, leading to an overproduction of reactive oxygen and nitrogen species [4, 5]. The other known mechanism of radiation injury, corresponding to an increased apoptosis, although not directly demonstrated by histology, can be hypothesized by observing ‘long-term’ the changed population of myocytes, newly generated or remodeled. About this, we underline that any increased cellular death proportionally enhances the release of inflammatory cytokines, providing also a chemo-attracting and mitogen action, so activating the fibroblasts and myoblasts normally present in the striated muscles; in this, we cannot exclude the possible co-participation of stem cells or a process of remodeling of the surviving myocells. In fact, the myocells that we observed ‘long-term’ demonstrated a spindle-like shape feature, which invites considering these cells as newly generated, rather than remodeled, ones that lined up according to the forces axes still present in the perineal floor. In all this, we see the promoting action of transforming growth factor alpha 1, promoting cellular necrosis and apoptosis, and then of other growth factors, e.g. vascular endothelial growth factor and fibroblast growth factor [6, 7]. Equally, the exuberant and often misguided reparative fibrosis, as observed ‘long-term’, was performed by a mature dense collagen connective tissue of the first type that interposed within the muscle fibers and wrapped their fine nervous structures, without an equivalent component of the extracellular matrix [8].

These observations agree with the difficult healing often observed after abdominoperineal rectal resections and explain some features of the ‘Lower Anterior Resection Syndrome’, which can be referred to damage of the perineal muscles and to their vasculonervous perineal structures, followed by an exuberant fibrosis [9, 10]. Clearly, our results have to be considered as preliminary, missing observations at intermediated times that are difficult to obtain due to the current scarcity of adequate surgical specimens; however, they can increase interest in this pathology and its clinical impact.

## Conflicts of interest

None declared.
